# Co-occurrence of liver metastasis of gastrointestinal stromal tumor and hepatocellular carcinoma: a case report

**DOI:** 10.1186/s40792-016-0212-z

**Published:** 2016-09-01

**Authors:** Kohei Yamashita, Yoshifumi Baba, Junji Kurashige, Masaaki Iwatsuki, Katsunori Imai, Daisuke Hashimoto, Yasuo Sakamoto, Akira Chikamoto, Naoya Yoshida, Toru Beppu, Hideo Baba

**Affiliations:** Department of Gastroenterological Surgery, Kumamoto University Graduate School of Medical Sciences, 1-1-1 Honjo, Chuo-ku, Kumamoto City, 860-8556 Japan

**Keywords:** GIST, HCC, Liver metastasis

## Abstract

Gastrointestinal stromal tumors (GISTs) are potentially malignant mesenchymal tumors that can give rise to distant metastases, mainly in the liver. The co-occurrence of synchronous primary liver tumors (e.g., hepatocellular carcinoma (HCC)) in patients with GIST is extremely rare. This report describes a 77-year-old male patient with liver metastasis of GIST originating in the small intestine and synchronous HCC. The patient had undergone resection of the small intestine for the primary GIST 3 years earlier and partial hepatectomy and radiofrequency ablation for liver metastases of GIST 1 year earlier. Despite the continuation of adjuvant therapy with imatinib, two new lesions in the liver were detected by follow-up computed tomography scanning, which showed the gradual enlargement of one tumor. A second hepatectomy was performed. Pathological examination revealed that one tumor was a liver metastasis of GIST and the other was a primary HCC. To our knowledge, this is the first report of the synchronous co-occurrence of a liver metastasis of GIST and a primary HCC.

## Background

Gastrointestinal stromal tumors (GISTs) are rare mesenchymal tumors of the gastrointestinal tract, arising mainly in the stomach and small intestine. GISTs were identified as tumors of the interstitial cells of Cajal, or their precursors, with more than 75 % showing mutations in KIT, thus defining a specific sarcoma subtype [1]. The availability of antibodies to KIT (CD117) has made the diagnosis easier [2]. Distant metastases of GIST arise predominantly in the liver and peritoneum, but rarely in the lymph nodes, bones, lungs, and other sites [3, 4]. Although GISTs have been reported to occur together with other malignancies, including chronic lymphocytic leukemia, lymphoma, renal cell carcinoma, and gastric cancer [5], few reports have described the synchronous occurrence of primary GISTs and hepatocellular carcinoma (HCC). To our knowledge, there have been no reports to date of the co-occurrence of a liver metastasis of GIST and primary HCC in the liver of individual patients.

## Case presentation

**Fig. 1:**
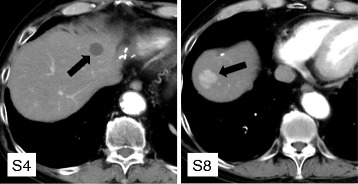
CT appearance of two liver tumors, in segments 4 and 8

**Fig. 2:**
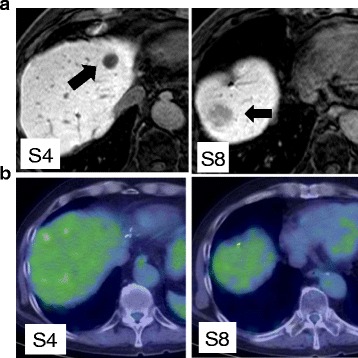
**a** Gd-EOB-DTPA-enhanced MRI appearance of two liver tumors, in segments 4 and 8. **b** PET-CT appearance of two liver tumors, in segments 4 and 8

**Fig. 3:**
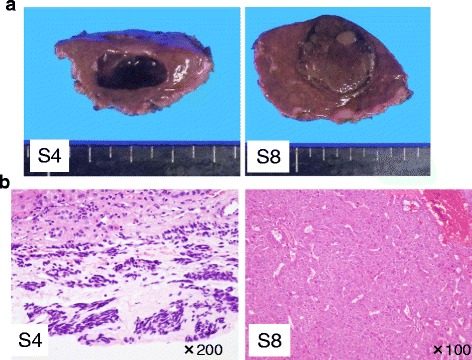
**a** Resected specimen containing the two liver tumors. **b** GIST with immunoexpression of KIT, hepatocellular carcinoma (H&E)

Three years ago, a 77-year-old male patient was admitted to our hospital with mild upper abdominal pain and mild nausea. Small intestinal fiber and computed tomography (CT) scanning showed a tumor in the small intestine and two tumors in the liver, a 22 × 11 mm nodular lesion in segment 3 and a 15 × 10 mm nodular lesion in segment 7. An endoscopic biopsy was obtained from the tumor in the small intestine, with pathological results compatible with GIST (KIT+; vimentin+). Imaging findings of the two liver tumors were consistent with metastases of GIST, and he was diagnosed with GIST originating in the small intestine and liver metastases of GIST. Because he had abdominal symptoms, he first underwent resection of the small intestine for the primary lesion. KIT mutation analysis of the resected tumor specimen showed an in-frame deletion of a portion of the juxtamembrane domain (exon 11). He was subsequently started on treatment with imatinib for the metastatic lesions. However, he experienced skin eruptions, a side effect of imatinib treatment. Eight months after resection of the small intestine, he underwent partial hepatectomy for the tumor in segment 3 and radiofrequency ablation (RFA) for the tumor in segment 7. A follow-up CT scan 5 months later showed tumor recurrence in segment 4 of the liver. He was again started on treatment with low-dose imatinib, during which the metastatic lesion became gradually enlarged, although the internal CT image showed a cystic change. In addition, a new lesion with arterial enhancement was detected in segment 8 of the liver. CT findings of these two tumors differed markedly, in that the S8 tumor showed enhancement in the arterial phase, while the S4 tumor did not (Fig. 1). Even in the hepatobiliary phase of Gd-EOB-DTPA-enhanced MRI, S8 tumor exhibited an inhomogeneous image while S4 tumors exhibited a well-demarcated low-density image (Fig. 2a). There were no abnormal uptakes on FDG-PET in the liver (Fig. 2b). As the patient had never pointed out the infection of hepatitis virus and other liver pathogens, tumor markers such as AFP and PIVKA-II were not measured. Our preliminary diagnosis was that both tumors were liver metastases of GIST. As increasing the dose of imatinib was problematic because of the side effects in this patient, a second hepatectomy was performed to remove these two lesions (Fig. 3a). Histologically, the lesion in segment 4 was a liver metastasis of GIST with necrotic changes, whereas the lesion in segment 8 was a primary HCC (Fig. 3b). Follow-up for 12 months after the latest surgery has shown no signs of recurrent disease.

## Discussion

Distant metastases of GIST arise predominantly in the liver and peritoneum. For example, of 80 patients with primary GIST who underwent complete resection, 63 % had recurrences in the liver, with the liver being the only site of disease in 44 % of patients with recurrences. A similar pattern was observed in 94 patients with metastasis at presentation, in that 65 % had liver lesions and 53 % had isolated liver recurrence. Hepatic tumors appearing after surgery for GIST should therefore be regarded as liver metastases of GIST. Importantly, the imaging findings of liver metastases of GIST vary and can be altered by treatment with molecularly targeted agents such as imatinib. Thus, subjective evaluations, including changes in tumor nodules, density, and vascularization, as well as changes in tumor size, are optimal in evaluating therapeutic responses by CT [6].

Patients with GIST frequently experience synchronous malignant tumors. For example, the frequency of malignant neoplasms was significantly higher among patients with GIST (22 %) than in the general population (4 %) [7]. Carcinomas of the gastrointestinal tract are the most frequent neoplasms associated with concomitant GIST, with gastric and colorectal adenocarcinomas being most prevalent [5]. Synchronous primary liver tumors have been reported in few patients with GIST, including one patient with a perivascular epithelioid cell tumor of the liver [8] and three with HCC (one KIT-positive and two KIT-negative) [9–11]. To our knowledge, this is the first report of the co-occurrence of a liver metastasis of GIST and a primary HCC in the liver.

The 5-year overall survival rate of isolated liver metastasis of GIST with hepatectomy was reported as 50 % [12], which was not poorer than that of early-stage HCC [13]. However, the recurrence rate following surgical resection for hepatic metastases from GIST was reported as high as 70–77 % [14]. Strict follow-up is needed in such described case.

## Conclusion

In conclusion, the co-occurrence of liver metastases of GIST and primary HCC is extremely rare. Because of differences in their treatment, synchronous hepatic lesions in patients diagnosed with GIST should be characterized by obtaining independent biopsies. This may aid in the correct and tailored treatment for individual patients.

## Consent

Written informed consent was obtained from the patient for publication of this case report and any accompanying images. A copy of the written consent is available for review by the Editor-in-Chief of this journal.
